# Elastic Modulus Measurement at High Temperatures for Miniature Ceramic Samples Using Laser Micro-Machining and Thermal Mechanical Analyzer

**DOI:** 10.3390/ma17184636

**Published:** 2024-09-21

**Authors:** Zhao Zhang, Hai Xiao, Rajendra K. Bordia, Fei Peng

**Affiliations:** 1 Department of Materials Science and Engineering, Clemson University, Clemson, SC 29634, USA; zzhang729@gmail.com; 2 Holcombe Department of Electrical and Computer Engineering, Clemson University, Clemson, SC 29634, USA; haix@clemson.edu

**Keywords:** picosecond laser, micro-machining, high-temperature ceramics, flexural elastic modulus, alumina, aluminum nitride

## Abstract

In this paper, we demonstrate a method of measuring the flexural elastic modulus of ceramics at an intermediate (~millimeter) scale at high temperatures. We used a picosecond laser to precisely cut microbeams from the location of interest in a bulk ceramic. They had a cross-section of approximately 100 μm × 300 μm and a length of ~1 cm. They were then tested in a thermal mechanical analyzer at room temperature, 500 °C, 800 °C, and 1100 °C using the four-point flexural testing method. We compared the elastic moduli of high-purity Al_2_O_3_ and AlN measured by our method with the reported values in the literature and found that the difference was less than 5% for both materials. This paper provides a new and accurate method of characterizing the high-temperature elastic modulus of miniature samples extracted from representative/selected areas of bulk materials.

## 1. Introduction

Accurately measuring the elastic modulus is vitally important for understanding material properties and engineering design [1]. Structural ceramics have high melting points, chemical stability, and mechanical strength. They are often used in high-temperature, corrosive environments and under substantial mechanical loads. Therefore, it is important to accurately measure the elastic modulus at high temperatures. The flexural resonance method [2,3,4,5] has been used to measure the elastic modulus up to 1600 °C. Based on the specific cross-section dimensions of the bar sample, the elastic modulus can be determined by the flexural and longitudinal mechanical resonance frequencies [6]. However, the resonance method is conducted on bulk or large-scale samples. Therefore, it cannot be used to measure the local elastic moduli of heterogeneous materials or composite materials with a non-uniform structure. To study the elastic modulus at selective locations of a product, researchers developed several microscale mechanical testing methods, including nanoindentation [7,8,9], the bulge test [10], and the in situ TEM/SEM micropillar compression test [11,12]. The challenges associated with these methods are sample preparation, handling, the application of small forces, stress and strain measurement, and conducting tests at elevated temperatures [13]. In terms of length scale, there is a gap between the bulk scale samples and the nano/micro samples. Thus, in this paper, we aim to demonstrate a straightforward method for characterizing the high-temperature, flexural elastic modulus of ceramics at selected locations for miniature samples at the millimeter scale.

The relationship between the elastic modulus and temperature is rather complicated. It involves changes in the binding energy due to temperatures and the volume change [14]. At very low temperatures, it is observed that the elastic constant changes with T^4^ [15]. At high temperatures, a linear relationship is observed for the elastic modulus for refractory oxides, including MgO, Al_2_O_3_, MgSiN_2_, Si_3_N_4_, and AlN [16,17,18,19]. The data can be fit to an empirical relationship proposed by Wachtman [18]:

(1) $$E=E_{0}-BT\cdot exp\left(-\frac{T_{0}}{T}\right)$$

where *E*_0_ is the elastic modulus at 0 *K*; *E* is the elastic modulus at an elevated temperature *T*; and *B* and *T*_0_ are both fitting parameters. For *T* ≫ *T*_0_, *E* ≈ *E*_0_ − *B*(*T* − *T*_0_), showing a linear relationship [3]. However, above a critical temperature, the elastic modulus for some ceramics has been found to decrease sharply with temperature due to grain boundary sliding and internal friction [6,9].

## 2. Flexural Test for Measuring Elastic Modulus

Although it is possible to measure the high-quality modulus data of metallic materials from the tensile test focusing on the low-strain part of the stress–strain curve [20], it is generally not feasible to use the same method to achieve an accurate measurement for ceramic materials due to their brittleness and the difficulty of making tensile samples and attaching them to testing machines. Instead, the elastic modulus, for ceramics, is generally measured using the flexural test (static methods) or dynamic methods (sound velocity). Compared with tensile tests, flexural testing in three- or four-point bending is able to achieve much larger displacement with smaller forces [21,22].

**Figure 1:**
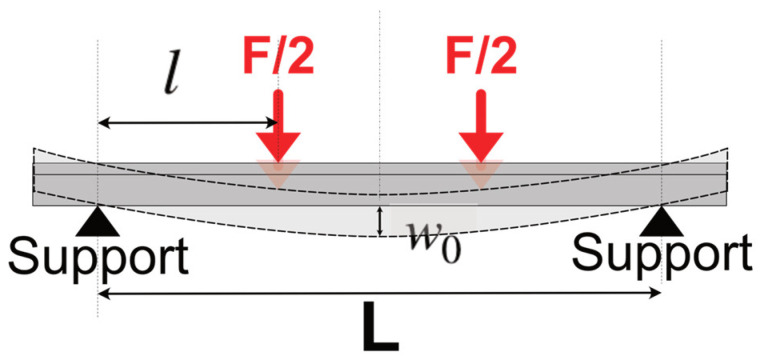
Four-point bending test geometry.

In a four-point flexural test (Figure 1), the deflection $w_{0}$ in the center of the beam is given by [23]:

(2) $$w_{0}=\frac{Fl(3L^{2}-4l^{2})}{48EI}$$

where *F*/2 is the force applied symmetrically at two locations of the test beam; *L* is the distance between two outer supports; *l* is the distance between the inner loading point and the outer support; and *I* is the geometrical moment of inertia of the beam’s cross-section.

With the dimensions of the sample and test set-up, the elastic modulus of the test sample can then be calculated as [23]:

(3) $$E=\frac{Fl(3L^{2}-4l^{2})}{48w_{0}I}$$

In this paper, we describe a method for measuring the elastic modulus of miniature ceramic samples. The test is based on a four-point flexural test, uses a thermal mechanical analyzer (TMA, Seko TMA SS6000, Hitachi High-Tech Analytical Science, Westford, MA, USA), and laser machining to make miniature samples from bulk samples. Using the picosecond laser, ceramics can be cut into microbeams with cross-sectional dimensions in the range of ~100 μm. Due to the small dimensions, TMA, which has a relatively low load capability, can be used to perform the flexural test at high temperatures. This process allows the measurement of the elastic modulus for miniature samples and also the local modulus of samples extracted from a large part, in which the structural variations are in the range of the sample size. It therefore bridges a relevant length scale—between bulk samples and local measurements at the scale probed by nanoindentation.

## 3. Experimental Procedure

### 3.1. Experimental Set-Up

**Figure 2:**
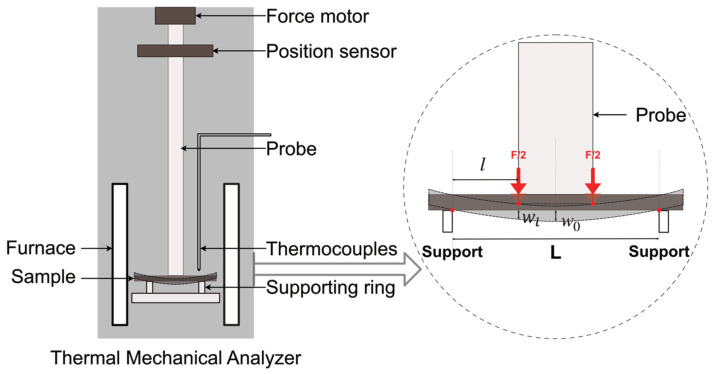
Elastic modulus measurement set-up for microbeam in TMA.

The experimental set-up is shown in Figure 2. The key factor in obtaining the elastic modulus successfully was the location of the microbeam in the center of the furnace and the placement of the probe symmetrically in the center of the microbeam. The supporting ring was made of high-purity alumina with an inner diameter of *L* = 8.8 mm. The diameter of the loading probe was 3.4 mm.

High-purity alumina (>99.6%, MTI Cooperation, Richmond, CA, USA) substrates with dimensions of 2″ × 2″ × 0.5 mm were purchased. The grain size and surface roughness were reported by the supplier to be less than 1 µm and 25 nm, respectively. A high-purity aluminum nitride substrate with dimensions of 1″ × 1″ × 0.5 mm (>99%, MTI Cooperation, Richmond, CA, USA) was also purchased. The surface roughness was reported by the supplier to be less than 10 nm.

**Figure 3:**
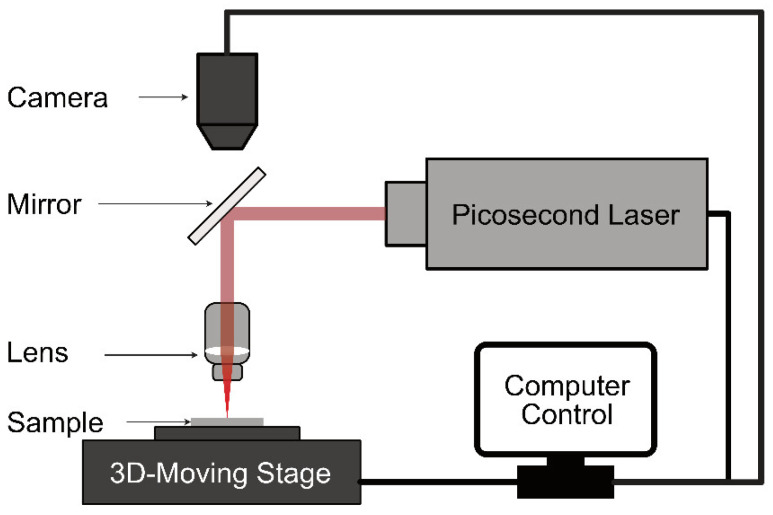
Picosecond laser set-up for laser-machined microbeams from ceramics.

**Figure 4:**
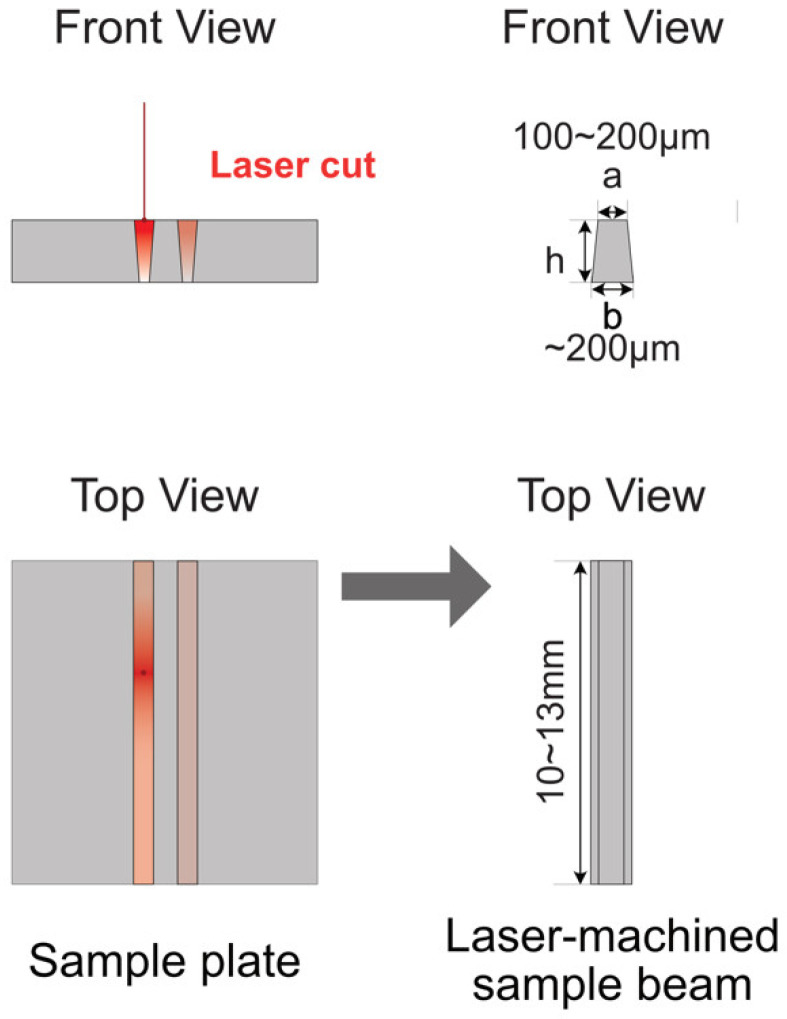
Geometry of ceramic samples before and after laser machining. In the front view, ‘a’ means the width of top surface, and ‘b’ means the width of the bottom surface.

**Figure 5:**
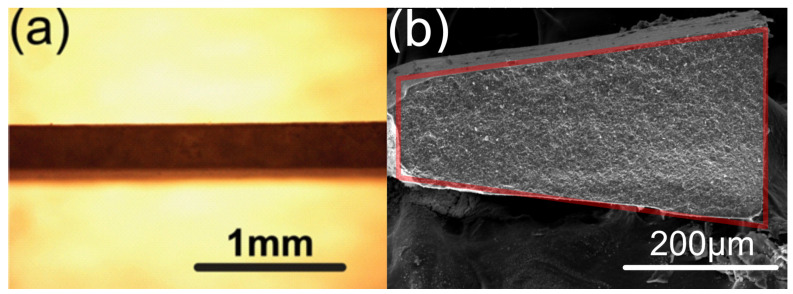
(**a**) Optical microscopy image of the top view of the microbeam; (**b**) SEM image of the cross-section of the microbeam, with an overlay of the ideal symmetrical trapezoidal shape for comparison.

For laser machining, the ceramic substrates were attached to a 3D moving stage (Figure 3). The distance between the lens and the sample substrate was adjusted to ensure the picosecond laser was focused on the top surface of the substrate to start with. The sample substrate moved at a controlled programmed speed to make the cut. Two parallel cuts were performed at the same time to ensure the uniformity of the microbeam. Depending on the laser power and substrate, repeating the procedure 20 or more times was needed before the focal point of the picosecond laser moved deeper into the substrate. The laser focal step along the *z* axis also depends on the laser power and substrate. A typical value in our set-up was 100 µm. Using this set-up, the dimensions of a laser-machined microbeam can be controlled. For our sample, the typical dimensions are shown in Figure 4, where h is the thickness of the starting substrate. It is important to note that the cross-section of the beam is trapezoidal due to the interaction of the laser with the sample and beam divergence. Figure 5a is an optical image of the top view of the laser-machined microbeam showing a uniform thickness microbeam. Figure 5b is an SEM image of the cross-section clearly showing the trapezoidal cross-section of the beam.

### 3.2. Laser-Machined Microbeam and Moment of Inertia

**Figure 6:**
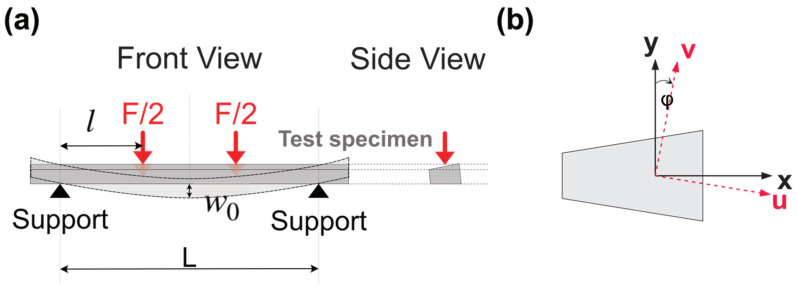
(**a**) The orientation of microbeam on TMA; (**b**) axes’ rotation for the moment of inertia calculation.

The loading geometry and the sample geometry are shown in Figure 6. The second moment of inertia of the trapezoidal cross-section beam sample for the *x* and *y* axis (Figure 6b) can be calculated by [24]:

(4) $$I_{x}=\frac{h^{3}(a^{2}+4ab+b^{2})}{36}$$

(5) $$I_{y}=\frac{h(a+b)(a^{2}+b^{2})}{48}$$

Considering the axes’ rotation (Figure 6b), the moment of inertia needs to be modified by the relation [24]:

(6) $$I_{v}=\frac{I_{x}+I_{y}}{2}-\frac{I_{x}-I_{y}}{2}cos\;2\varphi +I_{xy}sin\;2\varphi \;$$

In an isosceles trapezoid, with known dimensions of *a*, *b*, and *h*, the rotation angle *φ* can be calculated [25]:

(7) $$\varphi =arctan\;\frac{b-a}{2h}$$

If we assume the trapezoid is symmetrical, *I_xy_* = 0. Then, the moment of inertia of the beam sample for the *v* axis can be calculated using Equation (6).

Here, we need to mention that the vertical deflection measured by TMA is the deflection $w_{l}$ at the contact point at the edge of the probe (Figure 2). The maximum vertical deflection $w_{0}$ in the center of the beam can be calculated based on the deflection profile along the beam [24] and the deflection detected by TMA, $w_{l}$, using the geometric parameters shown in Figure 6a:

(8) $$\frac{w_{l}}{w_{0}}=\frac{12lL-16l^{2}}{3L^{2}-4l^{2}}$$

### 3.3. Load and Temperature Program

**Figure 7:**
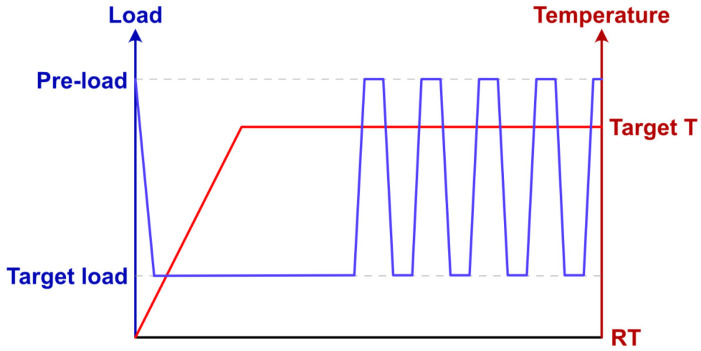
Load and temperature profile of an elastic modulus measurement.

Measurements were made at room temperature, 500 °C, 800 °C, and 1100 °C. The microbeam was placed in the center of the supporting ring, with the TMA probe applying a pre-load of −40 mN (the negative sign means the loading was compressive) on the microbeam. The supporting ring and the TMA probe were concentric. A target load of −200 mN was realized by increasing the load at a rate of 200 mN/min. This target load was applied by the probe of TMA and held. Then, the furnace temperature was raised to the target temperature at 10 °C/min and held for 1 h (for temperature equilibration) before the unloading–loading cycle started. A minimum of five loading and unloading cycles were conducted (between a load of −200 mN and −40 mN). A typical load and temperature profile is shown in Figure 7.

## 4. Results and Discussion

The dimensions of the microbeams, as shown in Table 1, were precisely measured using an optical microscope and SEM at multiple points. These samples were fabricated through the laser machining process described earlier. The observed variations in the trapezoidal cross-sectional dimensions between samples result from both the material-specific interactions with the laser and the setting of the gradual adjustments in the laser focus during each cutting cycle, which correspond to the increasing depth of the cut.

**Figure 8:**
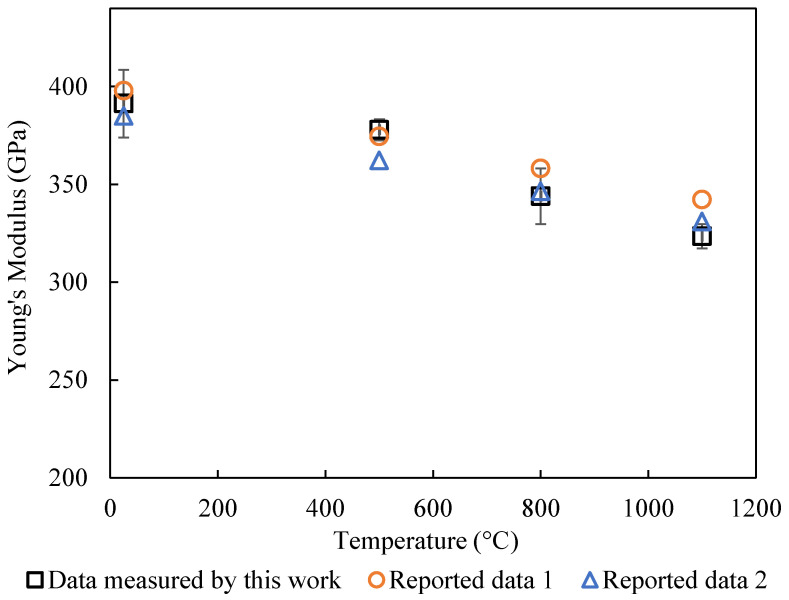
Comparison of the temperature dependence of elastic modulus for Al_2_O_3_ between experimental data and reported data in ref [16].

**Figure 9:**
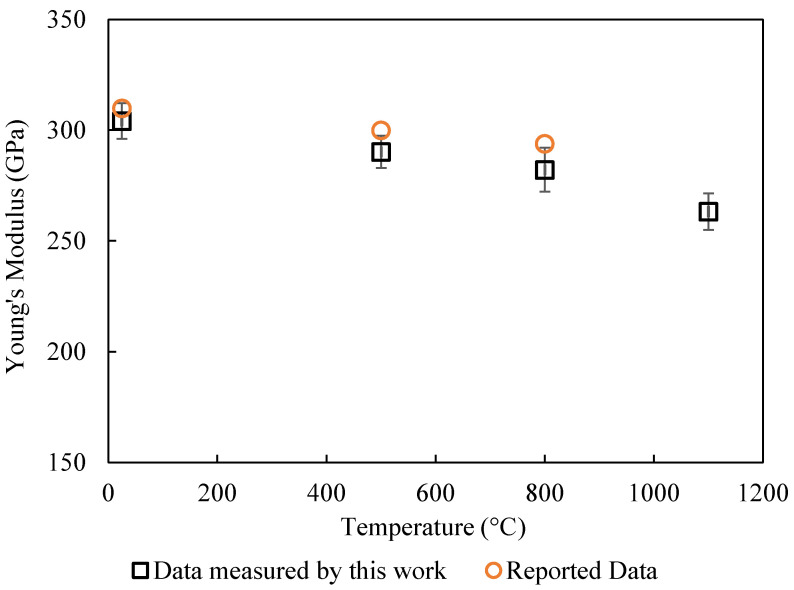
Comparison of the temperature dependence of elastic modulus for AlN between experimental data and reported data in ref [3].

The elastic modulus was calculated using Equation (3), in which the center point deflection, *w*_0_, is given by Equation (8) and the moment of inertia, *I_v_*, is given by Equation (6). The measured values of the elastic modulus are shown in Table 2 and Figure 8 and Figure 9. The measurement only focused on the initial portion of the stress–strain curve, where the sample behaved more as a linear elastic solid. The temperature dependence of the elastic modulus is almost linear for both alumina and aluminum nitride. This agrees with Wachtman’s theory [18]. The elastic modulus at 1100 °C was about 82% of the modulus at 25 °C. The results for the elastic modulus also agree with the reported data [3,16] within 5%, though uncertainty values were not provided in the cited references.

The unloading and loading cycles were set to start after the target temperature was reached and held for 1 h. This was done to equilibrate the temperature and hence the sample dimensions. The high loading rate reduced the TMA drift. Five loading/unloading cycles were performed for each run to obtain multiple measurements. The first cycle typically was not stable. This was caused by the “settling-in” of the test piece into the support ring [1].

Based on Equations (2) and (4)–(6), the magnitude of the deflection will decrease sharply with an increase in the cross-section of the microbeam. When the values of *a* and *b* are close, *φ* is close to one, and the deflection of the microbeam will be proportional to

$$\frac{1}{h(a+b)(a^{2}+b^{2})}$$

To introduce a high deflection of the sample for a given load, one needs to control the dimension of the microbeam, especially the thicknesses *a* and *b*. Based on the limitation of TMA and the high modulus of ceramic microbeams, typical dimensions of the beam, which can lead to significant deflection (significantly above the displacement resolution) are *a* =100 μm, *b* = 200 μm, and *h* = 500 μm. Because the measured elastic modulus is very sensitive to the dimensions of the microbeam, extra attention needs to be paid to carefully measuring the dimensions of the microbeam. Even though microbeams were laser-machined into the same size under the same conditions, the dimensions of each sample need to be determined individually. Using an optical microscope and SEM, the top width *a* and bottom width *b* need to be checked along the entire length to ensure uniformity.

Before the test, a safe maximum testing load can be estimated based on the maximum stress in the sample during the test and the strength of the material. The maximum load during the test should be set high enough to introduce considerable deflection without introducing any micro-cracks. For our microbeams, a maximum load of 200 mN was used.

**Figure 10:**
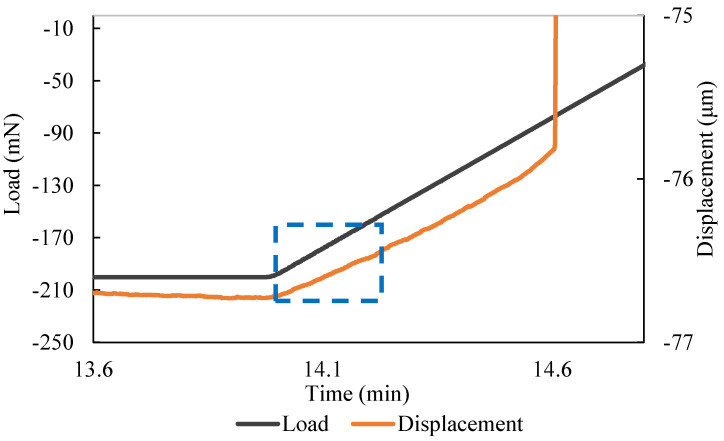
Part of data used to calculate sample’s elastic modulus is marked in the blue rectangle.

This baseline drift *w*_1_ is caused by the system. It can be corrected by replacing the test piece with a thick alumina plate. With the dimensions of the alumina plate, theoretically, the deflection would be neglectable. Therefore, the measured deflection can then be used to correct the baseline drift caused by the system. At high load (in the dotted rectangular area in Figure 10), the deflection is proportional to the load. The elastic modulus needs to be calculated in this region (the start of the unloading). In this load regime, the test piece is still “settled-in” in the fixture. When the load is low (the end of the unloading stage), the load and deflection are no longer proportional due to the movement of the microbeam.

**Figure 11:**
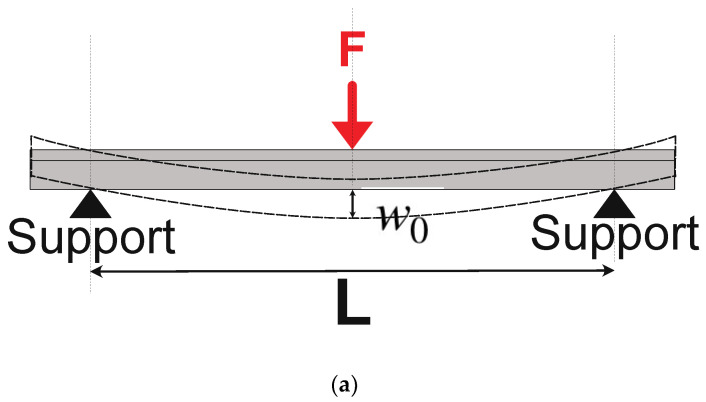

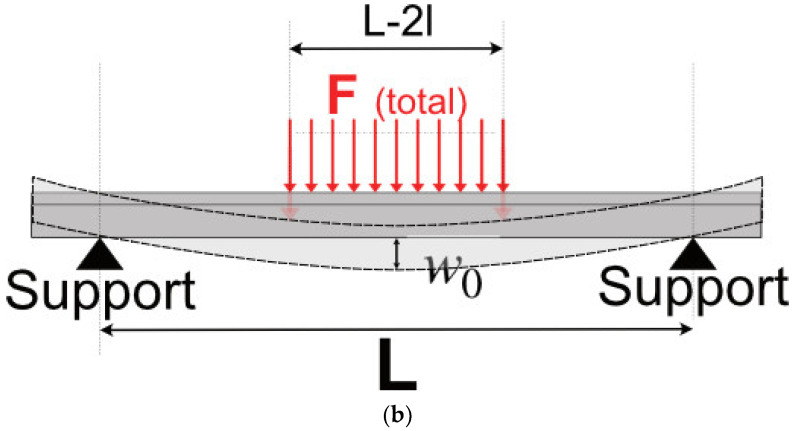
(**a**) Three-point bending test geometry. (**b**) Beam bending test with evenly distributed load.

Since the loading probe and the support rings are of finite width, which is not negligible compared to the beam length, the elastic modulus results, *E*_4*P*_, calculated based on the four-point flexural geometry (Figure 1), were compared with *E*_3*P*_, the results of the three-point geometry [24] (Figure 11a):

(9) $$E_{3P}=\frac{FL^{3}}{48Iw_{0}}$$

and *E_DL_*, the results of uniformly distributed-load beam [24] (Figure 11b):

(10) $$E_{DL}=\frac{F{(8L}^{3}-4{\left(L-2l\right)}^{2}L+{\left(L-2l\right)}^{3})}{384Iw_{0}}$$

**Figure 12:**
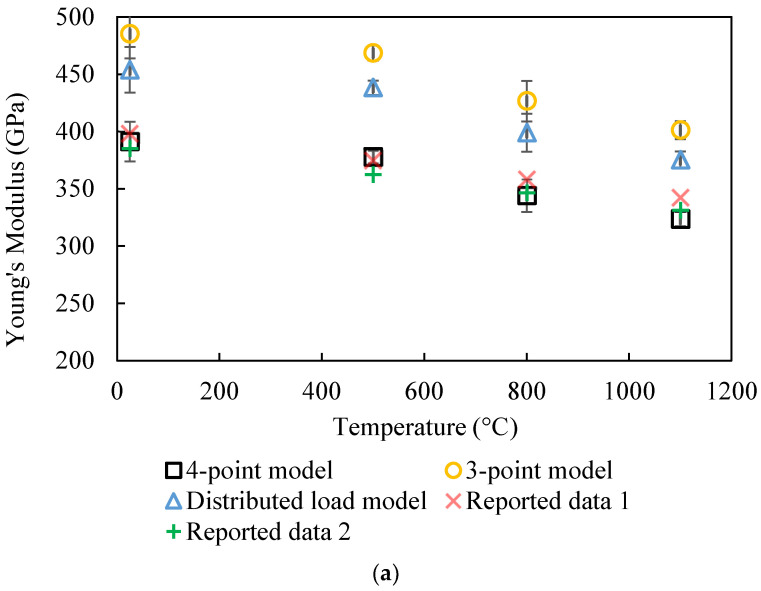

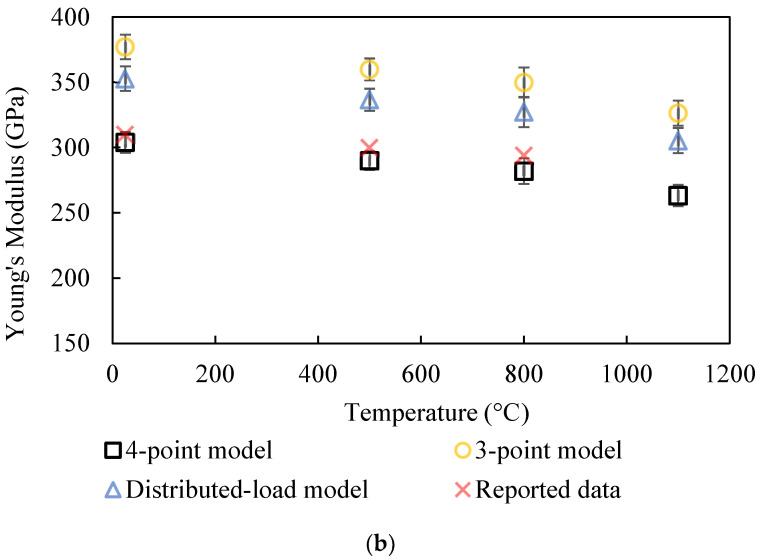
(**a**) Comparison of the temperature dependence of elastic modulus for Al_2_O_3_ between experimental results, using 4-point, 3-point, and distributed-load geometry, and reported values presented in Ref. [16]. (**b**) Comparison of the temperature dependence of elastic modulus for AlN between experimental results, using 4-point, 3-point, and distributed-load geometry, and reported values in Ref. [3].

The results of these three geometries are compared in Figure 12a for Al_2_O_3_ and Figure 12b for AlN. These results clearly demonstrate that the four-point geometry best describes the start of the unloading process during which the measurements were made. The three-point and distributed-load models resulted in higher Young’s modulus results, with an average factor of 1.24 times and 1.16 times compared to the values given by the four-point model, respectively (based on the specific dimensions of our set-up). This is because at the start of the unloading process, the microbeam had the highest displacement (*w*_0_). With the smooth surface of the microbeam processed by the picosecond laser, the TMA loading was only in contact with the microbeam at the perimeter of the probe of the TMA (Figure 2). Therefore, at the early stage of the unloading, the geometry can be treated as a four-point flexure test.

Table 3 compares elastic modulus measurement methods, highlighting their principles, advantages, and limitations. Traditional tests are easy but need bulk samples; resonance methods are non-destructive but sensitive to dimensions [1]. Nanoindentation and micropillar testing offer micron-scale measurements but face surface and fabrication challenges [7,8,9,11,12]. The novel method here provides localized, high-temperature measurements.

## 5. Conclusions

A new method of measuring the elastic modulus of ceramics at elevated temperatures based on TMA and laser-machined miniature beams has been developed. The test requires careful measurements of the geometry of the sample. The technique developed in this paper allows the investigation of the elastic modulus at a length scale that is in between the traditional macro-scale (sample sizes of cm and higher) and micro-techniques (sample size of 10 to 100 microns) at high temperatures. The length scale used in this test allowed us to both investigate the average properties of small samples and probe the localized properties in materials where the inhomogeneity is at the length scale of relevance for this test. The needed corrections to the raw data are discussed together with the different potential loading geometries. A comparison of the experimental results for the reported value of two ceramics shows very good agreement, giving a measure of confidence in the use of this technique.

## Figures and Tables

**Table 1:** The geometrical dimensions of alumina and aluminum nitride samples.

	Sample #	L (mm)	a (μm)	b (μm)	h (μm)
Alumina	1	8.80	97 ± 2	275 ± 6	500 ± 5
	2	8.80	97 ± 2	290 ± 7	500 ± 5
	3	8.80	71 ± 3	245 ± 6	500 ± 5
	4	8.80	115 ± 4	255 ± 9	500 ± 5
Aluminum nitride	5	8.80	157± 3	240 ± 6	500 ± 5
	6	8.80	97 ± 2	228 ± 4	500 ± 5
	7	8.80	89 ± 2	243 ± 2	500 ± 5

**Table 2:** The comparison of elastic moduli measured in this work and in the literature.

Temperature (°C)	Alumina (GPa)			Aluminum Nitride (GPa)	
	Report in Ref. [16]	Report in Ref. [16]	This Paper	Report in Ref. [3]	This Paper
25	398	385	391 ± 17	310	304 ± 8
500	374	362	378 ± 5	300	290 ± 7
800	358	347	344 ± 14	294	282 ± 10
1100	342	331	324 ± 6	-	263 ± 8

**Table 3:** Comparison of methods for measuring elastic modulus.

Method	Principle	Advantages	Limitations
Tensile and flexure tests	Measures deformation under applied stress	Easy to prepare; standardized and widely used	Bulk or large-scale samples
Resonance and impact excitation methods	Measures natural frequency or response to impact	Non-destructive; high-temperature capability	Bulk or large-scale samples; dimensional sensitivity; high surface finish requirement; suspension and support issues at high temperature
Nanoindentation	Measures indentation hardness and modulus using a sharp indenter	Localized measurements (micron-scale)	Sensitive to surface conditions; complexity in analysis
Micropillar testing	Measures compressing or deforming of small, cylindrical pillars	Localized measurements (micron-scale)	Fabrication challenges; small stress–strain measurement; complexity in analysis; properties may differ from those of bulk materials
This work	Measures deformation of laser-machined microbeam under applied stress with a TMA	Simple result analysis; high-temperature testing; easy control of inert atmosphere Localized measurements (millimeter-scale)	Requires precise set-up; requires laser micro-machining capability

## Data Availability

The original contributions presented in the study are included in the article, further inquiries can be directed to the corresponding author.
